# Determinants of immunoglobulin G responses to respiratory syncytial virus and rhinovirus in children and adults

**DOI:** 10.3389/fimmu.2024.1355214

**Published:** 2024-03-04

**Authors:** Alicia Guillien, Katarzyna Niespodziana, Marion Mauclin, Anne Boudier, Raphäelle Varraso, Bénédicte Leynaert, Orianne Dumas, Nicole Le Moual, Thomas Schlederer, Maja Bajic, Kristina Borochova, Peter Errhalt, Raphaël Vernet, Rachel Nadif, Jean Bousquet, Emmanuelle Bouzigon, Rudolf Valenta, Valérie Siroux

**Affiliations:** ^1^ University Grenoble Alpes, Inserm U 1209, CNRS UMR 5309, Team in Environmental Epidemiology Applied to Development and Respiratory Health, Institute for Advanced Biosciences, Grenoble, France; ^2^ Division of Immunopathology, Department of Pathophysiology and Allergy Research, Center for Pathophysiology, Infectiology and Immunology, Medical University of Vienna, Vienna, Austria; ^3^ CHU Grenoble Alpes, Grenoble, France; ^4^ Université Paris-Saclay, UVSQ, Université Paris-Sud, Inserm, Equipe d’Epidémiologie Respiratoire Intégrative, Centre de recherche en Epidémiologie et Santé des Populations (CESP), Villejuif, France; ^5^ Karl Landsteiner University, Krems, Austria; ^6^ Department of Pneumology, University Hospital, Krems, Austria; ^7^ Université Paris Cité, Inserm, UMRS 1124, Group of Genomic Epidemiology of Multifactorial Diseases, Paris, France

**Keywords:** immunoglobulin G, respiratory syncytial virus, rhinovirus, adults, children

## Abstract

**Introduction:**

Exposure to respiratory viruses is a significant cause of morbidity and affects virus-specific antibody levels. Little is known about determinants associated with immune response to these viruses. We aimed to investigate the determinants of respiratory syncytial virus (RSV)- and rhinovirus (RV)- specific IgG responses in both children and adults.

**Methods:**

The study is based on the EGEA cohort, composed of 530 samples of children in EGEA1 (1991-95) and 1241 samples of adults in EGEA2 (2003-07). Cumulative RV-specific IgG levels (species A, B and C) and IgG levels to RSV-G protein were measured by using micro-array technoloy. Multiple linear mixed models (random effect to account for familial dependence) were performed to assess associations between age, sex, body mass index (BMI), tobacco smoke exposure and season of blood sampling with RSV-and RV-specific IgG levels.

**Results:**

In children (11.1 ± 2.8 years old, 57% boys), higher RV-specific IgG levels were associated with older age (only for RV-B), female sex and lower BMI, while only older age was associated with higher RSV-specific IgG levels. In adults (43.5 ± 16.7 years old, 48% men), younger age, female sex, lower BMI, active smoking and all seasons except summer were associated with higher RV-specific IgG levels. Older age, active smoking and all seasons except summer were associated with higher RSV-specific IgG levels.

**Conclusion:**

Personal and seasonal determinants of RSV- and RV-specific IgG levels seem to vary according to the respiratory virus type and between children and adults, suggesting different patterns of responses along the life course.

## 1 Introduction

Exposure to respiratory viruses is a significant cause of morbidity worldwide both in children and adults. Among children, respiratory syncytial virus (RSV) causes bronchiolitis and is the most common cause of respiratory hospitalization and the second biggest cause of lower respiratory infection mortality worldwide (1). Other respiratory viruses, especially rhinovirus (RV) strains, are also major risk factors of respiratory morbidity because they are known to trigger severe asthma exacerbations in children (2). Longitudinal studies showed that RSV and RV-induced bronchiolitis and wheezing illness in early childhood are associated with subsequent development of asthma (3–8). In healthy adults, RSV and RV are responsible of common colds, with frequent reinfections; and in frail elderly persons, they cause insidious deteriorations of respiratory health with high mortality (2). Additionally, RV can trigger symptoms of asthma and asthma exacerbations in allergic patients and individuals susceptible to viral-induced attacks (9).

Infections of RSV and RV viruses are more common during childhood. Virulence and persistence of specific immune responses to these respiratory viruses differ between individuals. To date, the studies identifying determinants, in particular personal factors (age, sex, body mass index (BMI), tobacco smoking) and season of blood sampling, of RSV- and RV-specific antibody response remains scarce and often conflicting results are reported, especially related to passive and active tobacco exposure (10–12). Moreover, none of these studies focused on populations including both children and adults.

Using micro-array technology, it is possible to measure RSV-specific antibody response as well as RV-specific antibody responses to different RV species (13, 14). Accordingly, cumulative levels of RV-specific antibody levels may be considered as an immunological imprint of previous RV infections and their severity. However, there may be also differences regarding the severity of RV infections depending on the RV-species involved. For example, among the three RV species, RV-A and RV-C were associated with more severe illness and RV-B with mild symptoms or asymptomatic infections, especially in children (15, 16).

In the present study, we performed a comprehensive analysis to assess RSV and RV-specific IgG responses in a large cohort of well characterized children and adults to identify factors associated with RSV and RV-specific antibody responses. For the accurate measurement of RV- and RSV-specific antibody levels we used defined RV peptides and recombinant RSV-derived G protein which were immobilized by micro-array technology on chips. Peptides and antigens were spotted in triplicates to assure accurate determinations of antibody levels (13, 14). Furthermore, RV peptides (17) and recombinant RSV G protein (18) have been shown in earlier studies to bind high levels of virus-specific antibodies.

## 2 Methods

### 2.1 Study population

**Figure 1:**
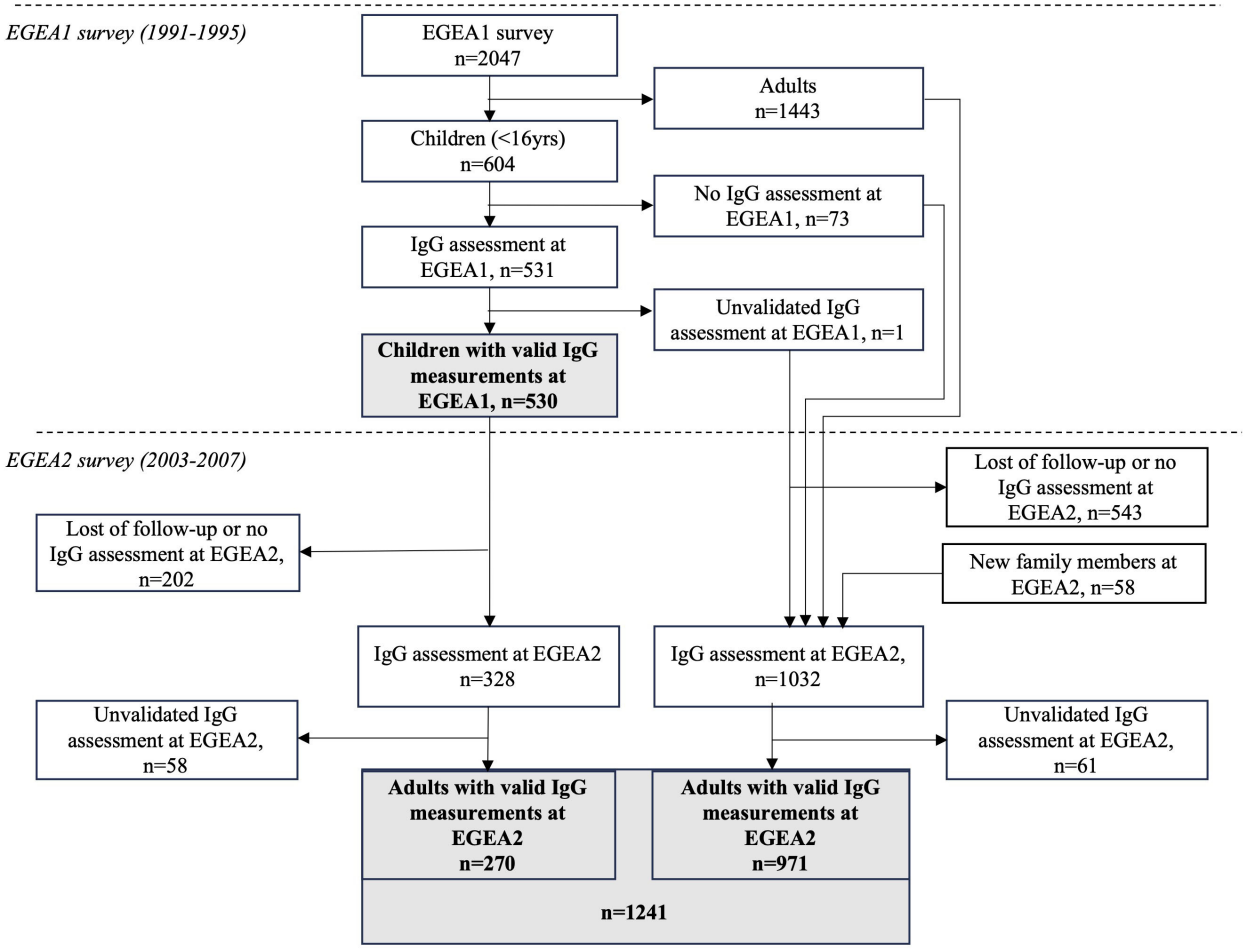
Flow-chart of measurements performed in the study population. EGEA, Epidemiological study on the Genetics and Environment of Asthma; IgG, Immunoglobulin G; EGEA1, baseline survey (1991-95). EGEA2: first follow-up of the EGEA participants (2003-07), about 12.5 years after EGEA1. Measurements for virus-specific IgG levels were performed by microarray technology. Unvalidated IgG assessment corresponds to samples excluded due to the lack of sufficient sample volume.

The study is based on the Epidemiological study on the Genetics and Environment of Asthma (EGEA) cohort, which combines a group of asthma cases recruited in chest clinics with their first-degree relatives and a group of population-based participants (**Figure 1**). At baseline (EGEA1), 2047 individuals (both children and adults) were recruited in 1991-1995 in five French cities. A first follow-up (EGEA2) was set-up in 2003-2007, including 1845 individuals from EGEA1 and 58 new family members. Virus-specific IgG responses were measured in 2021 in a subsample of 531 children (530 with valid measure) from the EGEA1 survey and 1360 adults (1241 with valid measure) from the EGEA2 survey. Among them, 329 individuals (270 with valid measure) had IgG responses measured in both EGEA1 and EGEA2 surveys. Written informed consent was obtained from all adult participants and child’s legal guardians at both surveys; and ethical approvals were obtained from the ethics committees (Cochin Royal Hospital, Paris for EGEA1 and Necker-Enfants Malades Hospital, Paris for EGEA2).

### 2.2 Microarrays containing synthetic RV-VP1 N terminal peptides, recombinant RSV-G protein

A detailed description of microarray procedure is provided in the **Supplementary Materials**. Comprehensive lists of the chip components spotted on microarrays are reported in **Supplementary Tables S1-2**. Synthetic VP1 N terminal peptides representing the three genetic RV species (RV-A: n=18; RV-B: n=9; RV-C: n=10) were selected and produced as described in previous studies (13, 14). The recombinant G protein of the RSV A2 strain described in (18) was expressed in *Escherichia coli* BL21 (DE3) instead of insect cells. Food allergens used for the calibration of the microarrays were purchased from Sigma Aldrich (Saint Louis, MO, USA) (**Supplementary Table S2**). Glass slides containing six microarrays surrounded by an Epoxy frame were obtained from Paul Marienfeld GmbH & Co. KG (Lauda-Königshofen, Germany) and coated with an amine-reactive complex organic polymer, MCP-2 (Lucidant Polymers, Sunnyvale, CA, USA). Microarray components were used at a concentration of 1 or 2 mg/ml in a phosphate buffer (75 mM Na_2_HPO_4_), pH = 8.4 and spotted in triplicates using a SciFlexArrayer S12 (Scienion AG, Berlin, Germany). After spotting, printed slides were placed in a humid chamber at 75% relative humidity and incubated at room temperature (RT) overnight. Slides were then blocked with 50 mM ethanolamine in PBS/Tween, pH=9, for 15 min and subsequently covered with a plate sealer (Candor, Wangen, Germany). Microarrays were vacuumed and stored at 4°C until use.

### 2.3 Measurement of virus-specific IgG using micro-array technology

For the measurement of virus-specific IgG antibodies, micro-arrays were processed as previously described (13, 19). Briefly, micro-arrays were first washed with phosphate-buffered saline (PBS) containing 0.1% Tween 20 (Sigma-Aldrich) for 5 min by stirring and dried by centrifugation (1 min, 1000 g, RT). Serum samples were diluted 1:300 using ImmunoCAP^®^ Specific IgA/IgG Sample Diluent (Phadia, Uppsala, Sweden) and 30 µl of diluted sera, a calibrator and sample diluent for each analysis run were applied onto each microarray and incubated for 2 hours at gentle rocking at room temperature (RT) (Biometra, Jena, Germany). Afterwards slides were washed again, dried by centrifugation and incubated 30 min with 30 µl/per array of affiniPure F(ab’)2 goat anti-huIgG (Jackson ImmunoResearch Laboratories, West Grove, PA, USA) labelled with DyLight 550 (Pierce, Thermo Fisher Scientific, Rockford, IL, USA). After further rinsing, washing and drying by centrifugation, microarrays were scanned with a confocal PowerScanner (Tecan Grödig, Austria) using 30% of gain (i.e., photomultiplier) and 10% of laser power. Scanned images were analysed using the Mappix software (Innopsys, Carbonne, France).

### 2.4 Management of virus-specific IgG data

All EGEA1 and EGEA2 samples were analyzed at the same time. **Supplementary Figure S1** shows a workflow representing the different steps of the management of IgG variables.

Fluorescence intensities (FI) of three replicated spots were measured and the median value of triplicate measurements was calculated (raw data). A calibration of raw data was performed, considering the calibrator and sample diluent estimated in each analysis run (calibrated data).

To determine the analytical sensitivity for each antigen, the background signals of all sample diluent repetitive measurements (n=21) were calculated as a mean FI values + 3 standard deviation for each antigen. Values below this background signal were zero (corrected data). For RV-specific IgG data, summary RV-A, RV-B and RV-C variables were calculated by the sum of IgG responses to 18, 9 and 10 specific peptides respectively, as listed in **Supplementary Table S1**.

### 2.5 Assessment of personal factors

For both EGEA1 and EGEA2 surveys, participants underwent a clinical visit and responded to a detailed questionnaire, based on international standardized tools (20). Potential personal determinants tested in the present study were: age at the time of IgG assessment, sex, BMI at the time of IgG assessment and passive and active tobacco smoking. Moreover, season of blood sampling (from which IgG responses to respiratory virus were measured) was also considered (“January-March”, “April-June”, “July-September” and “October-December”) because there is evidence for seasonal differences regarding respiratory virus infections (21, 22). In EGEA1, children were considered as exposed to passive smoking if they lived with at least one parent who was an active smoker at the time of the study. In EGEA2, adults were defined as “never smoker”, “former smoker” (daily smoker who stopped smoking for at least 1 month before the survey) or “active smoker” (current smoker of cigarettes, pipes, cigarillos or cigars at the time of the survey).

Ever-asthma was defined by a positive answer to the question “Have you ever had attacks of breathlessness at rest with wheezing?”, or “Have you ever had asthma attacks?”, or having being recruited as asthmatic case in a chest clinic in EGEA1. Allergic sensitization was defined by at least one positive result (mean wheal diameter of 3mm greater than that of the negative control) to the allergen extracts studied by skin-prick testing. Eleven allergens extracts were tested in the EGEA1 population (cat, *Dermatophagoides pteronyssinus*, *Cladosporium herbarum*, *Alternaria tenuis*, timothy grass, olive, birch, *Parietaria judaica*, ragweed, Aspergillus and *Blattella germanica*) and 12 in the EGEA2 population (the 11 tested in the EGEA1 population plus cypress).

Finally, presence of a respiratory infection few weeks prior to blood collection was defined by a positive answer to the question “Have you had a respiratory infection in the last 3 (4 in the EGEA2 population) weeks?”.

### 2.6 Statistical analyses

The reproducibility of the assay was assessed from sera of four individuals, which were measured in each analysis run (thus providing six replicates), by assessing the coefficient of variation (CV) and intraclass correlation coefficient (ICC) for each antigen.

For descriptive purpose, distributions of RSV- and RV-specific IgG levels in children and adults and across the different determinants studied (age, sex, BMI, tobacco smoking and season of blood sampling) were presented using box plots. To meet the assumptions of regression-based methods, RSV and RV-A/B/C were normalized by using the Ordered Quantile Normalization method (23) in the pooled EGEA1 and EGEA2 measured samples. In regression models, RSV- and RV-specific IgG levels were then expressed as z-scores and named zRSV, zRV-A, z-RV-B and zRV-C.

To assess the associations between potential determinants and IgG levels to RSV and RV-A/B/C, multiple mixed linear regression models considering age, sex, BMI, tobacco smoking (passive in children, active in adults) and season of blood sampling were performed, adjusted for ever-asthma and allergic sensitization. Family correlation was considered as a random effect. The analyses were performed separately in the EGEA1 and EGEA2 populations to assess these associations in children and in adults, respectively. A sensitivity analysis was conducted by performing this main analysis after exclusion of participants reporting having experienced respiratory infections within the three (four for adults from EGEA2) weeks before blood collection.

The main analysis was then performed with stratification on sex.

Similarly, the main analysis was performed with stratification regarding asthma status and allergic sensitization. Indeed, due to the design of EGEA cohort with a recruitment related to asthma, collider bias can be an issue when investigating, as in our study, associations between factors (some of the personal factors and RSV- and RV-specific IgG levels) that may both influence asthma and allergic sensitization. Results from separate analyses among participants with and without asthma and participants with and without allergic sensitization may inform about the presence of such a bias.

Finally, among individuals with RSV- and RV-specific IgG levels measured at both time points, associations between time elapsed during the two follow-ups and the evolution of IgG level over time was assessed using linear mixed models to account for repeated data, adjusted for age at first survey, sex, BMI, exposure to tobacco, season of blood sampling, allergic sensitization and ever-asthma.

## 3 Results

### 3.1 Description of the population

A total of 530 children from EGEA1 (mean (min-max) age: 11.1 (2–16); 57% boys) and 1241 adults from EGEA2 (mean (min-max) age 43.4 (18–79); 48% men) had valid IgG measurements and were analyzed in this study (**Figure 1**). Among them, 270 individuals had IgG measurements both in EGEA1 and EGEA2 surveys. The main characteristics of the included population are presented in **Table 1**. In EGEA1, 42% of the children were exposed to passive smoking. In EGEA2 half of the population was ever-smoker and almost half of the ever-smokers were still current smokers. The number of individuals with ever-asthma and allergic sensitization was 274 (52%) and 331 (64%) among children, respectively, and 498 (40%) and 648 (55%) among adults, respectively. Individuals with two IgG measures were younger at the second survey as compared to all EGEA2 participants (22.9 ± 3.1 years vs. 43.0 ± 17.0 years).

**Table 1:** Main characteristics of population under study.

	All participants included		Participants with longitudinal data (IgG measurements in EGEA1 and EGEA2)	
	EGEA1 - children	EGEA2 - adults	EGEA1 - children	EGEA2 - adults
	(n=530)	(n=1241)	(n=270)	(n=270)
Age (years)	11.1 ± 2.8	43.4 ± 16.7	11.2 ± 2.9	22.9 ± 3.1
Male, n (%)	303 (57%)	595 (48%)	142 (53%)	142 (53%)
BMI (kg/m2)	17.8 ± 2.9	24.4 ± 4.3	17.7 ± 2.7	22.2 ± 3.0
Exposure to passive smoking, n (%)	208 (42%)	–	93 (38%)	–
Tobacco smoking				
Never smoker, n (%)	–	630 (51%)	–	146 (54%)
Former smoker, n (%)	–	339 (27%)	–	27 (10%)
Active smoker, n (%)	–	270 (22%)	–	97 (36%)
Season of blood sampling				
January-March, n (%)	152 (29%)	301 (24%)	73 (27%)	56 (21%)
April-June, n (%)	112 (21%)	388 (31%)	50 (19%)	90 (33%)
July-September, n (%)	117 (22%)	224 (18%)	71 (26%)	44 (16%)
October-December, n (%)	149 (28%)	328 (26%)	76 (28%)	80 (30%)
Ever-asthma, n (%)	274 (52%)	498 (40%)	142 (53%)	160 (59%)
Allergic sensitization, n (%)	331 (64%)	648 (55%)	180 (67%)	201 (77%)
Respiratory infection in the past weeks*, n (%)	56 (11%)	124 (10%)	27 (10%)	31 (11%)

Results are presented as mean ± standard deviation or n (%).*Past 3 weeks for EGEA1 and past 4 weeks for EGEA2.

EGEA, Epidemiological study on the Genetics and Environment of Asthma; BMI, body mass index.

### 3.2 Reproducibility and distribution of RSV- and RV-specific IgG levels

**Figure 2:**
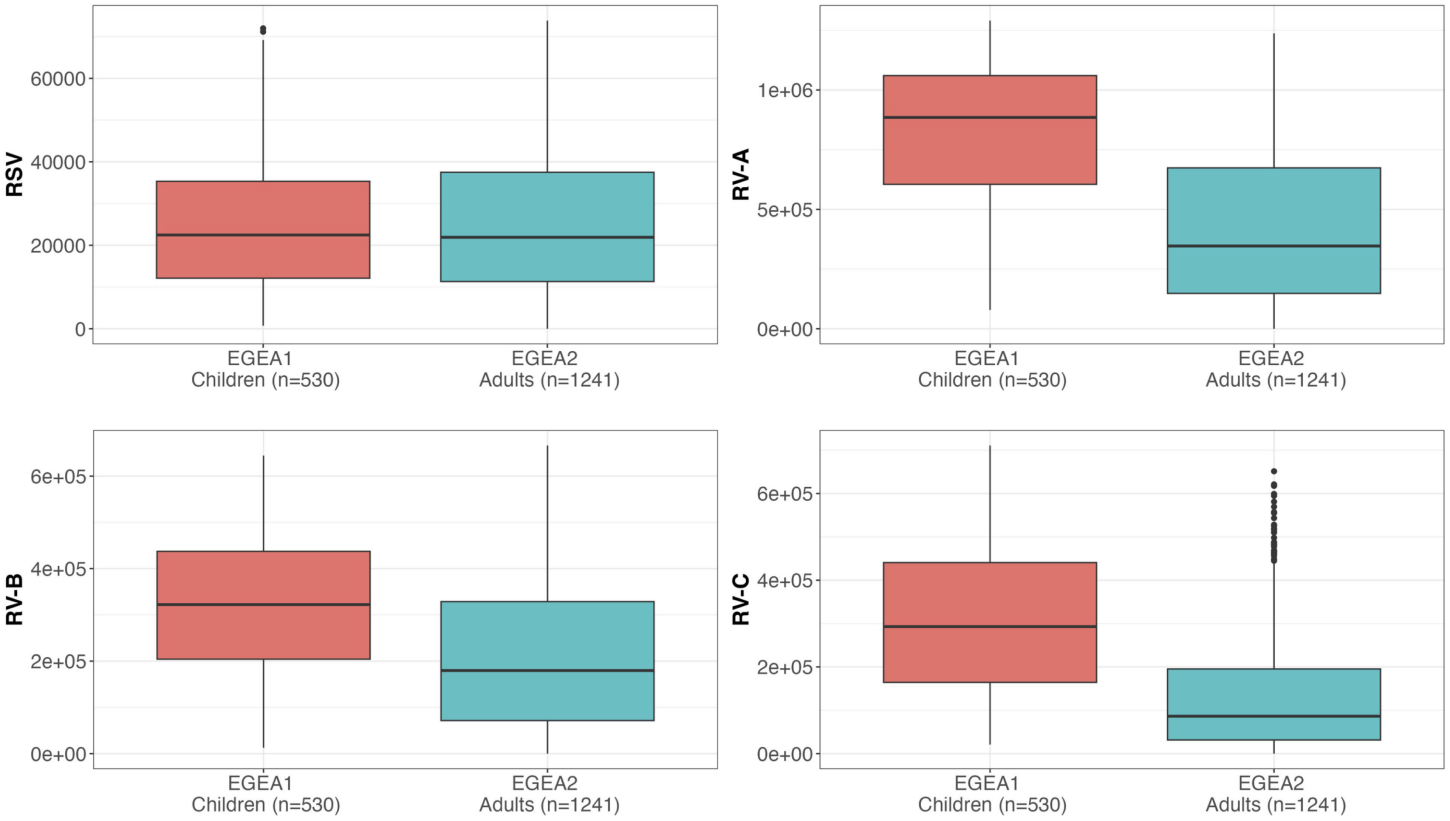
Distribution of RSV and RV-specific IgG levels in children (EGEA1, n=531) and adults (EGEA2, n=1241). Shown are IgG levels expressed as fluorescence intensity values (FI: y-axes) to RSV-G protein and VP1 N terminal peptides summed to represent RV-A, RV-B and RV-C species. Summary RV-A, RV-B and RV-C variables were calculated by the sum of IgG responses to 18, 9 and 10 specific peptides respectively, as listed in **Supplementary Table S1**. Data are shown as box plots (EGEA1: orange box plots; EGEA2: blue box plots). The lower and upper horizontal lines (hinges) correspond to the first and third quartiles (the 25th and 75th) percentiles. The horizontal line between the first and the third quartile correspond to the median. The upper whisker (vertical line) extends from the hinge to the largest value no further than 1.5*IQR from the hinge (where IQR is the inter-quartile range, or distance between the first and third quartiles). The lower whisker (vertical line) extends from the hinge to the smallest value at most 1.5*IQR of the hinge. Data beyond the end of the whiskers are called "outlying" points and are plotted individually. RSV, respiratory syncytial virus; RV, rhinovirus; EGEA, Epidemiological study on the Genetics and Environment of Asthma.

The reproducibility and distribution of RSV- and RV-A/B/C specific IgG levels are presented in **Table 2** and **Figure 2**. All values were above the background levels. Additionally, the distribution of IgG levels to each peptide under study are presented in **Supplementary Figure S2**. RV-specific IgG levels were higher among children from EGEA1 than in adults from EGEA2 for the three species, while RSV-specific IgG levels were similar in children and adults (**Figure 2**). Reproducibility was high with a mean CV at 0.12 for RV-specific IgG and 0.21 for RSV, and with excellent ICC (ICC ≥ 0.93 for all virus-specific IgG data). The correlations between IgG levels to RSV and to RV-A/B/C were low (r ≤ 0.27 in children and r ≤ 0.42 in adults), while the correlation was mild to strong between IgG levels across the three RV species (r=0.32 between RV-B and RV-C to 0.72 between RV-A and RV-C in children and r=0.53 to 0.81 in adults) (**Supplementary Figure S3**).

**Table 2:** Reproducibility, background level and distribution of virus-specific IgG levels .

	Reproducibility		Background level	Distribution in all participants included, median [IQR]		Distribution in participants with longitudinal data (measured in EGEA1 and EGEA2), median [IQR]	
	mean CV	ICC		EGEA1 - children	EGEA2 – adults	EGEA1- children	EGEA2 – adults
				(n=530)	(n=1241)	(n=270)	(n=270)
**RSV**	0.21	0.93	75.2	22473 [12110-35537]	21914 [11317-37496]	23065 [11831-36510]	18132 [8041-33241]
**RV-A**	0.12	0.97	67.1	885068 [604924-1060300]	346732 [11317-37496]	896054 [594349-1054170]	581326 [271219-895467]
**RV-B**	0.12	0.99	138.6	322210 [204258-437388]	179646 [71527-328715]	318204 [205738-444790]	217956 [114922-383097]
**RV-C**	0.12	0.97	74.2	293043 [164379-440619]	86343 [31524-195329]	281459 [158782-449288]	135255 [53365-305127]

Reproducibility was assessed from samples of 4 individuals of EGEA2 survey which were measured on each of the 6 arrays. For each virus-specific IgG level, the mean of the CV calculated for each of the four individuals is reported. Distributions of virus-specific IgG data are presented as median [interquartile range] of the calibrated and corrected for the background level values (before normalization) and expressed in FI.

IgG, Immunoglobulin G; CV, coefficient of variation; ICC, intra-class correlation coefficient; EGEA, Epidemiological study on the Genetics and Environment of Asthma; RSV, respiratory syncytial virus; RV, rhinovirus; FI, fluorescence intensity.

### 3.3 Factors that influence RSV- and RV-specific antibody levels

**Figure 3:**
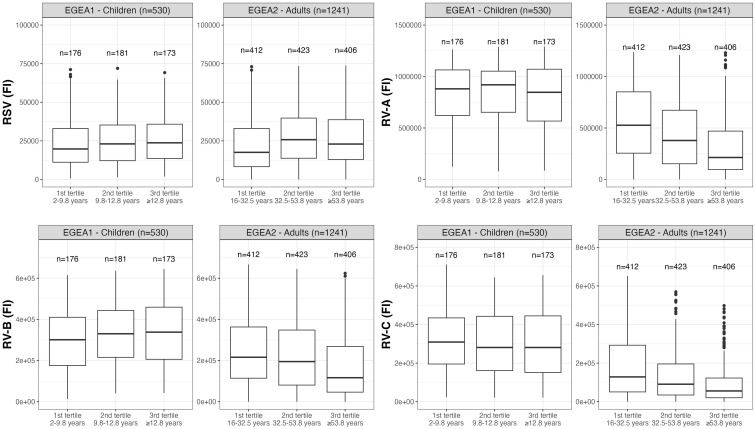
Distribution of RSV- and RV-specific IgG levels among tertiles of age in children (EGEA1, n=531) and adults (EGEA2, n=1241). Data expressed in fluorescence intensity (FI) values (y:axes) corresponding to RSV- and RV-A/B/C-specific antibody levels are shown as box plots. The lower and upper horizontal lines (hinges) correspond to the first and third quartiles (the 25th and 75th) percentiles. The horizontal line between the first and the third quartile correspond to the median. The upper whisker (vertical line) extends from the hinge to the largest value no further than 1.5*IQR from the hinge (where IQR is the inter-quartile range, or distance between the first and third quartiles). The lower whisker (vertical line) extends from the hinge to the smallest value at most 1.5*IQR of the hinge. Data beyond the end of the whiskers are called "outlying" points and are plotted individually. RSV: respiratory syncytial virus; RV: rhinovirus; EGEA: Epidemiological study on the Genetics and Environment of Asthma.

**Figure 4:**
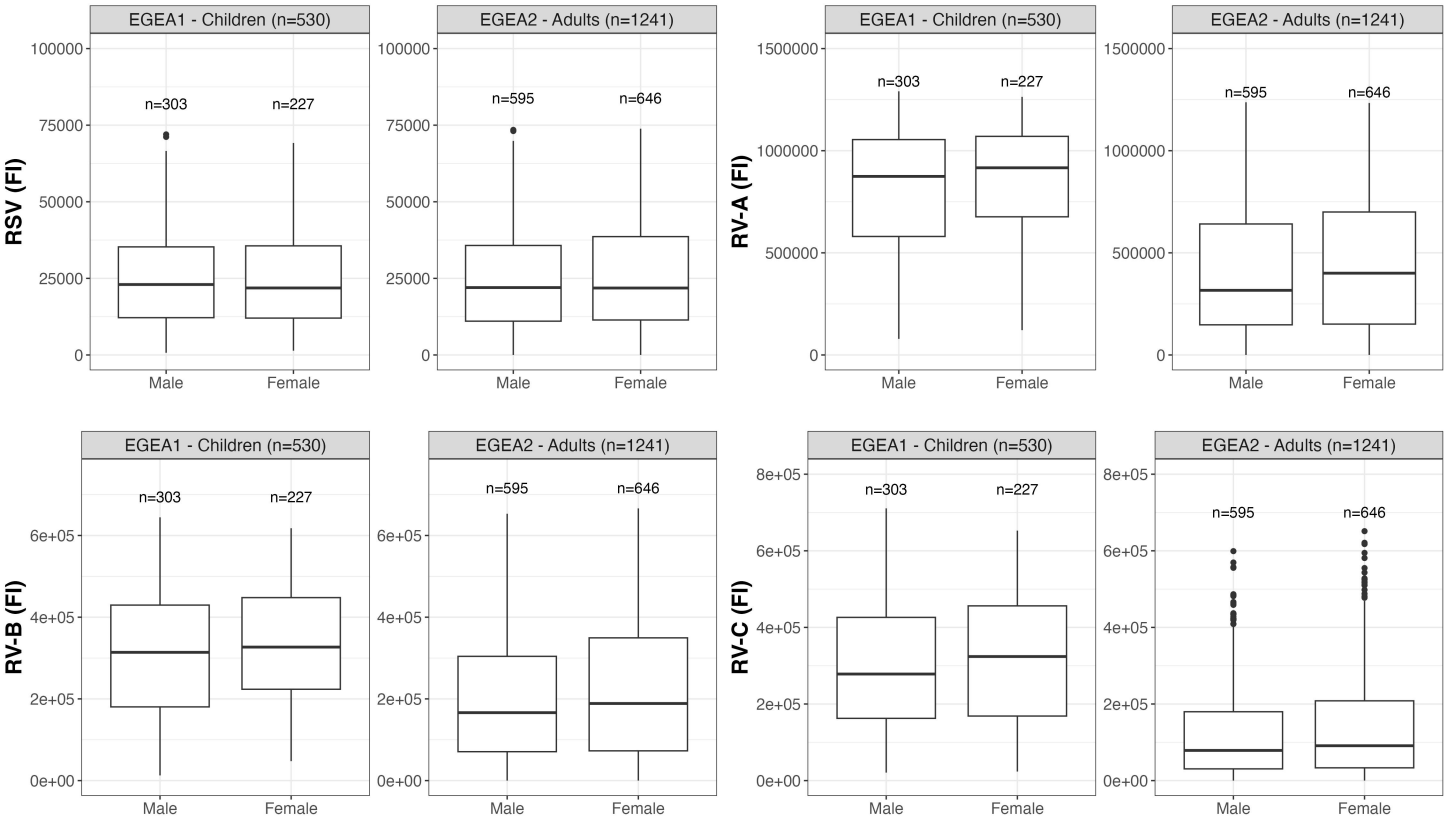
Distribution of RSV- and RV-specific IgG levels among male and female in children (EGEA1, n=531) and adults (EGEA2, n=1241). Data expressed in fluorescence intensity (FI) values (y:axes) corresponding to RSV- and RV-A/B/C-specific antibody levels are shown as box plots. The lower and upper horizontal lines (hinges) correspond to the first and third quartiles (the 25th and 75th) percentiles. The horizontal line between the first and the third quartile correspond to the median. The upper whisker (vertical line) extends from the hinge to the largest value no further than 1.5*IQR from the hinge (where IQR is the inter-quartile range, or distance between the first and third quartiles). The lower whisker (vertical line) extends from the hinge to the smallest value at most 1.5*IQR of the hinge. Data beyond the end of the whiskers are called "outlying" points and are plotted individually. RSV: respiratory syncytial virus; RV: rhinovirus; EGEA: Epidemiological study on the Genetics and Environment of Asthma.

**Figure 5:**
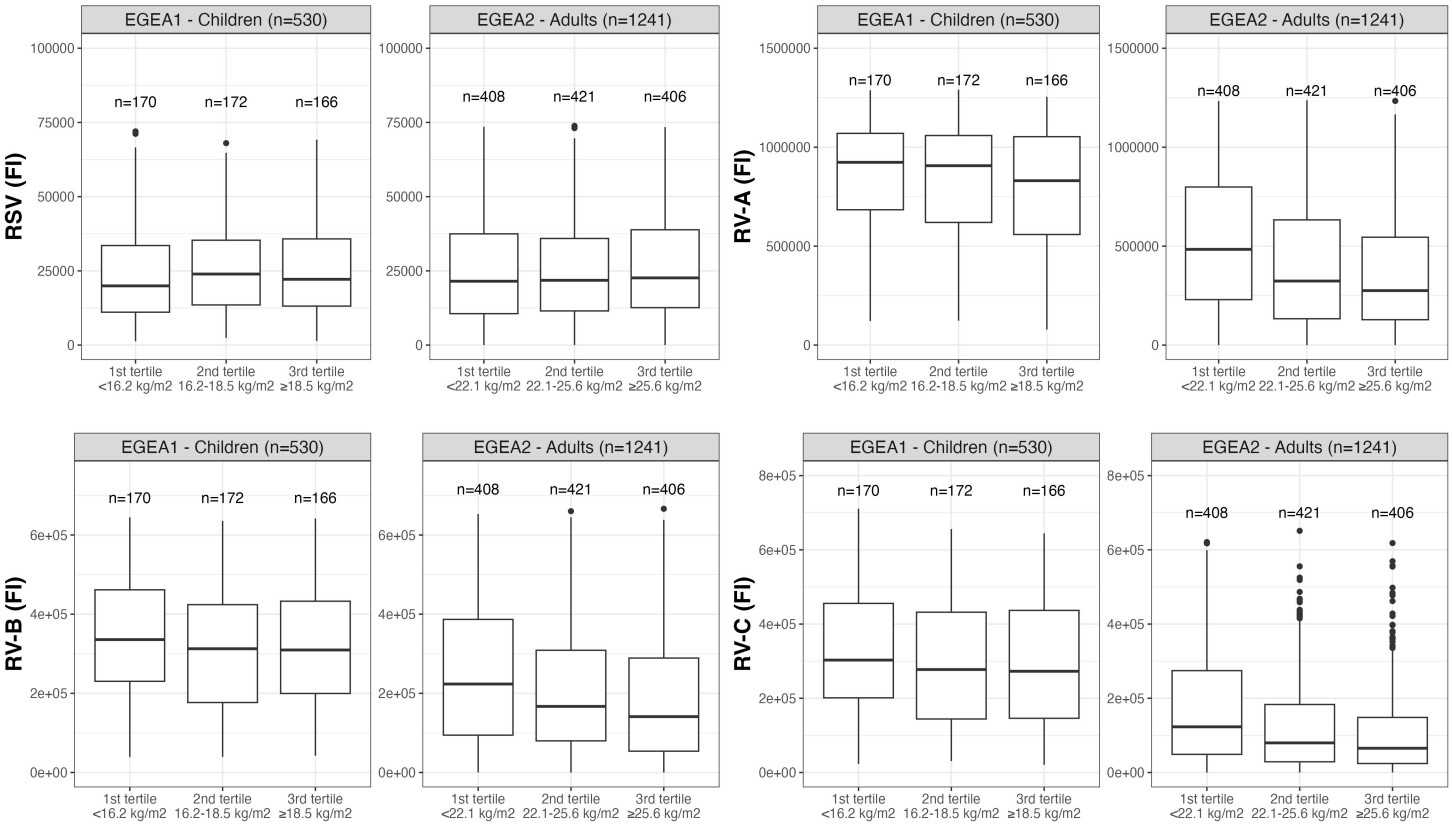
Distribution of RSV- and RV-specific IgG levels among tertiles of BMI in children (EGEA1, n=531) and adults (EGEA2, n=1241). Data expressed in fluorescence intensity (FI) values (y:axes) corresponding to RSV- and RV-A/B/C-specific antibody levels are shown as box plots. The lower and upper horizontal lines (hinges) correspond to the first and third quartiles (the 25th and 75th) percentiles. The horizontal line between the first and the third quartile correspond to the median. The upper whisker (vertical line) extends from the hinge to the largest value no further than 1.5*IQR from the hinge (where IQR is the inter-quartile range, or distance between the first and third quartiles). The lower whisker (vertical line) extends from the hinge to the smallest value at most 1.5*IQR of the hinge. Data beyond the end of the whiskers are called "outlying" points and are plotted individually. RSV: respiratory syncytial virus; RV: rhinovirus; EGEA: Epidemiological study on the Genetics and Environment of Asthma; BMI: body mass index.

**Figure 6:**
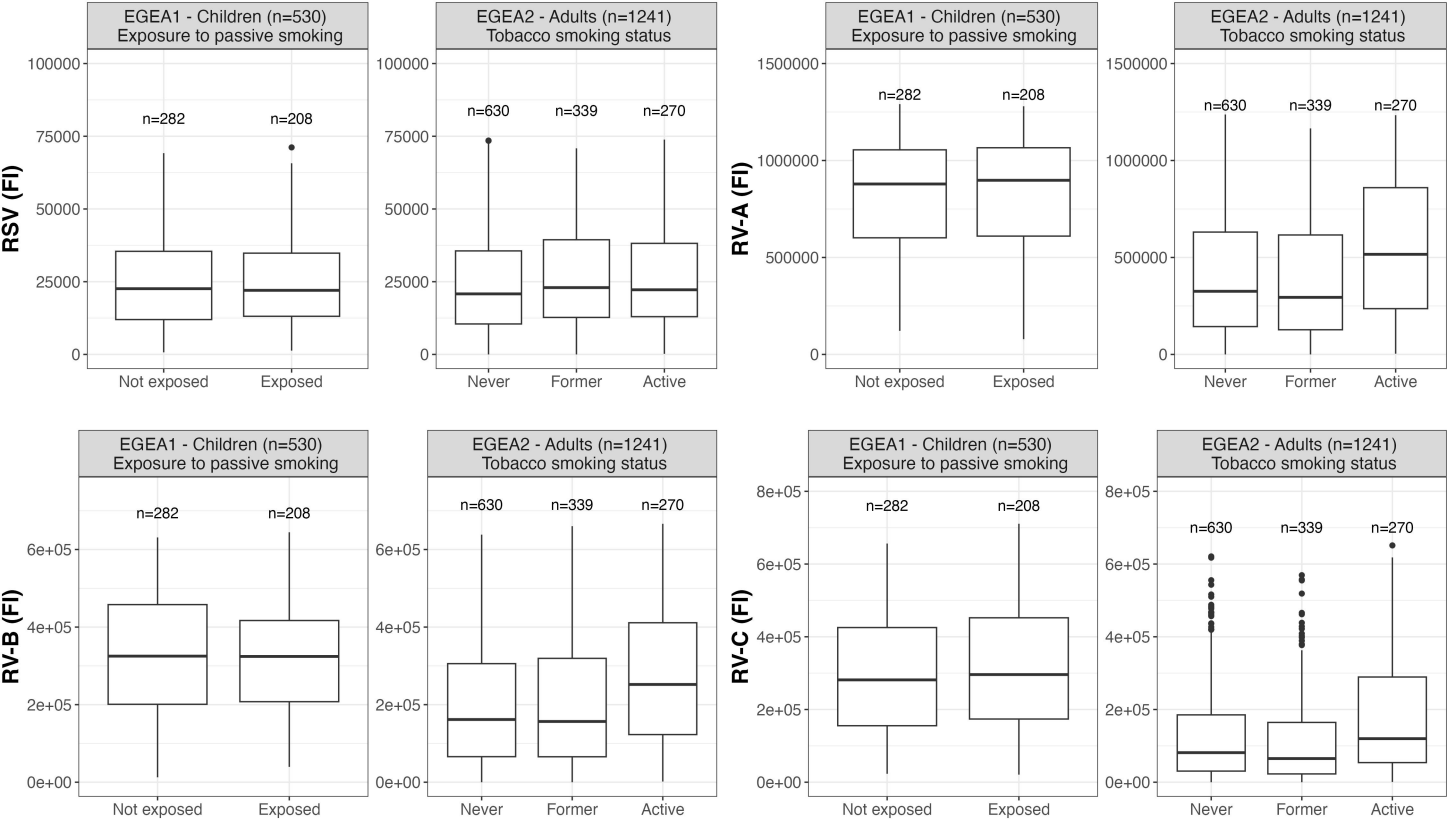
Distribution of RSV- and RV-specific IgG levels by passive smoking and tobacco smoking status in children (EGEA1, n=531) and adults (EGEA2, n=1241); Data expressed in fluorescence intensity (FI) values (y:axes) corresponding to RSV- and RV-A/B/C-specific antibody levels are shown as box plots. The lower and upper horizontal lines (hinges) correspond to the first and third quartiles (the 25th and 75th) percentiles. The horizontal line between the first and the third quartile correspond to the median. The upper whisker (vertical line) extends from the hinge to the largest value no further than 1.5*IQR from the hinge (where IQR is the inter-quartile range, or distance between the first and third quartiles). The lower whisker (vertical line) extends from the hinge to the smallest value at most 1.5*IQR of the hinge. Data beyond the end of the whiskers are called "outlying" points and are plotted individually. RSV: respiratory syncytial virus; RV: rhinovirus; EGEA: Epidemiological study on the Genetics and Environment of Asthma.

**Figure 7:**
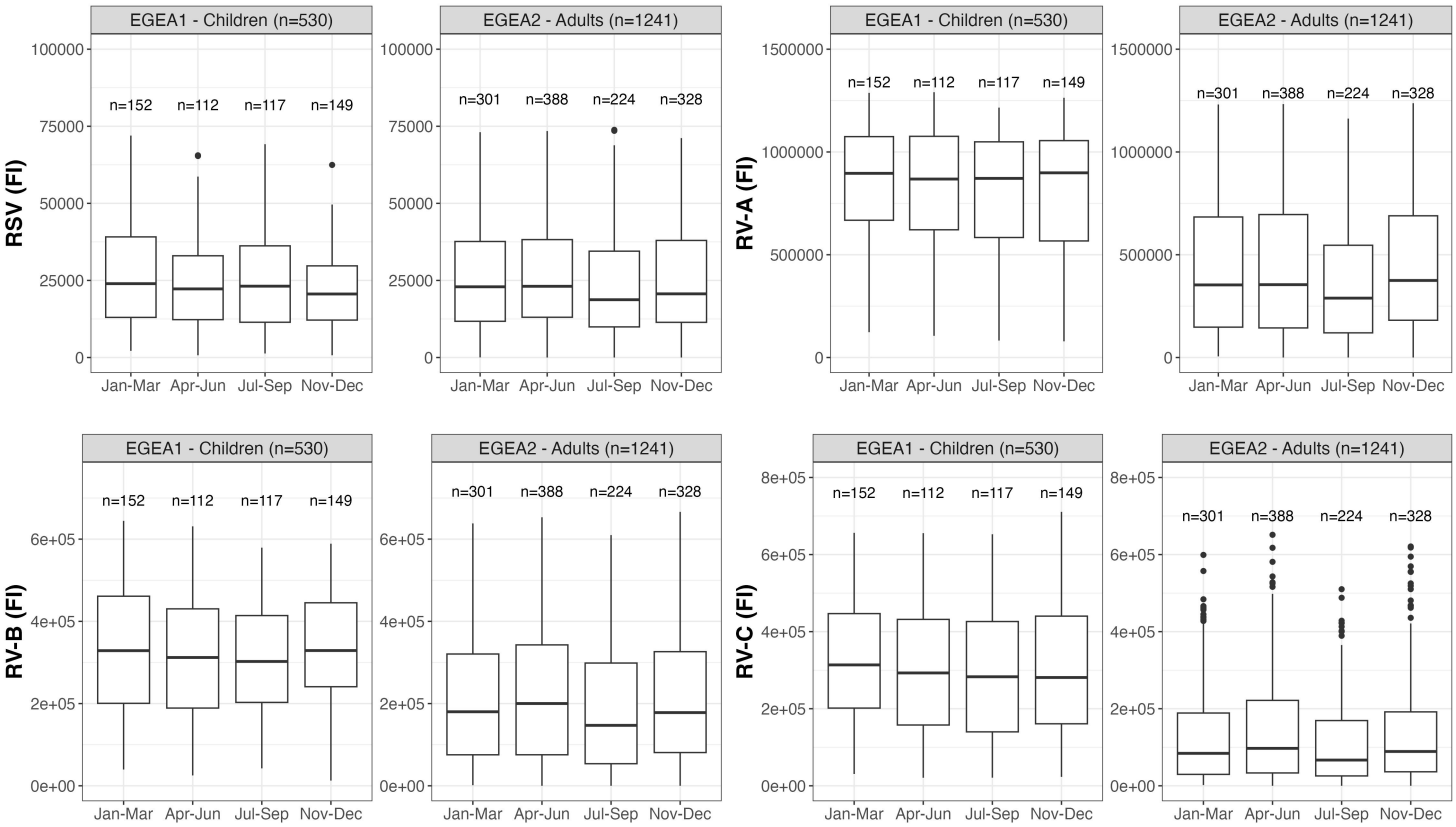
Distribution of RSV- and RV-specific IgG levels by seasons of blood sampling in children (EGEA1, n=531) and adults (EGEA2, n=1241); Data expressed in fluorescence intensity (FI) values (y:axes) corresponding to RSV- and RV-A/B/C-specific antibody levels are shown as box plots. The lower and upper horizontal lines (hinges) correspond to the first and third quartiles (the 25th and 75th) percentiles. The horizontal line between the first and the third quartile correspond to the median. The upper whisker (vertical line) extends from the hinge to the largest value no further than 1.5*IQR from the hinge (where IQR is the inter-quartile range, or distance between the first and third quartiles). The lower whisker (vertical line) extends from the hinge to the smallest value at most 1.5*IQR of the hinge. Data beyond the end of the whiskers are called "outlying" points and are plotted individually. RSV: respiratory syncytial virus; RV: rhinovirus; EGEA: Epidemiological study on the Genetics and Environment of Asthma; Jan: January; Mar: March; Apr: April; Jun: June; Jul: July; Sep: September; Nov: November; Dec: December.

Distributions of RSV- and RV-specific IgG levels among tertiles of age (**Figure 3**), sex (**Figure 4**), tertiles of BMI (**Figure 5**), tobacco exposure categories (**Figure 6**) and seasons (**Figure 7**) are presented in both children and adults.

**Figure 8:**
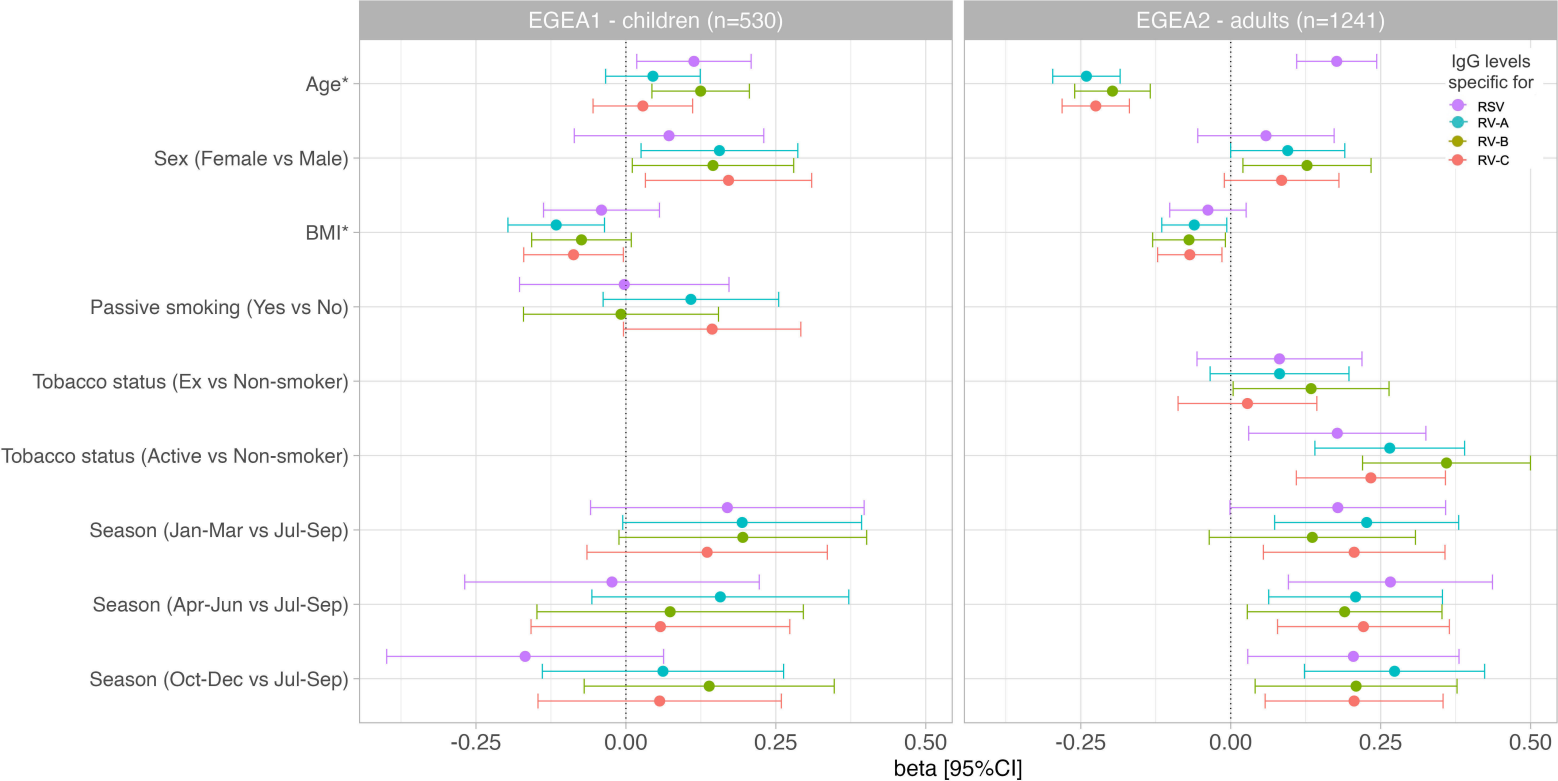
Associations of RSV and RV-specific IgG levels with personal determinants and seasons of blood sampling in children (EGEA1, n=531) and adults (EGEA2, n=1241). Multivariable mixed linear regression models were used to estimate beta and corresponding 95% CIs for virus-specific (RSV/RV) antibody levels associated with personal determinants and the seasons of blood sampling. Error bars represent 95% CIs. EGEA, Epidemiological study on the Genetics and Environment of Asthma; RSV, respiratory syncytial virus; RV, rhinovirus; BMI, body mass index; Jan-Mar, January-March; Apr-Jun, April-June; Jul-Sep, July-September; Oct-Dec, October-December. Beta were estimated from linear mixed regression models for repeated data (random effect on family) with IgG level as dependent variable and age (continuous), sex, BMI (continuous), season of blood sampling (4 seasons), tobacco (passive in EGEA1, active in EGEA2), allergic sensitization and ever-asthma as predictors. *Age and BMI were standardized (divided by their Standard deviation (SD)), thus beta are reported for 1 SD increased. SD of age in EGEA1: 2.85 years; SD of age in EGEA2: 16.7 years; SD of BMI in EGEA1: 2.91 kg/m^2^; SD of BMI in EGEA2: 4.25 kg/m^2^.

Results of the mutually-adjusted association study are presented in **Figure 8** and **Supplementary Table S3**. Among children in EGEA1, an older age was significantly associated with higher RSV- and RV-B- specific IgG levels (p=0.02 and 0.003, respectively). Female sex and lower BMI were associated with higher RV-A/B/C-specific IgG levels but sex and BMI were not associated with RSV-specific IgG levels. Passive smoking and season of sampling were not associated with RSV- nor RV-specific IgG levels (**Figure 8** and **Supplementary Table S3**).

Among adults in EGEA2, an older age was associated with higher RSV-specific IgG levels, and conversely with lower RV-specific IgG levels. Overall, females had higher RV-specific IgG levels than males (significant association with RV-B and trend for an association with RV-A and RV-C). Higher BMI was associated with lower levels of RV-specific IgG, but not with RSV-specific IgG levels. Former smokers, and in a greater extent current smokers had higher IgG levels to RSV- and RV than never-smokers. Samples collected in January to June and October to December showed higher RSV- and RV-specific IgG levels than samples collected from July to September (**Figure 8** and **Supplementary Table S3**).

In total, 56 (11%) children and 124 (10%) adults reported a respiratory infection within the three (four for adults) weeks before blood collection (**Table 1**). Distributions of RSV- and RV-specific IgG levels were similar between individuals with and without respiratory infection. Among the population of children and adults who did not report respiratory infection prior to blood collection, associations with personal and seasonal factors were very similar to associations reported in the whole population (**Supplementary Figure S4**).

Among children, the associations between older age and higher level of RV-B specific IgG and between higher BMI and lower RSV- and RV-specific IgG levels were found only in boys (**Supplementary Figure S5**). Among adults, overall similar patterns of associations were found in men and women, but with stronger magnitude of associations for smoking, BMI and season among men and for age among women (**Supplementary Figure S6**).

Overall, all these associations were found separately among individuals with and without ever-asthma, except in children for the associations between RSV-specific IgG levels and age and BMI which were specifically observed among children with asthma (**Supplementary Figures S7, S8**). Regarding allergic sensitization, all estimates reported in the whole populations of children and adults were similar in the subpopulation of children and adults with and without allergic sensitization (**Supplementary Figures S9, S10**), with wider confidence intervals, certainly due to the smaller sample sizes, which implies a reduction in statistical power.

### 3.4 Eleven-year individual change of respiratory virus-specific IgG levels, from childhood to early-adulthood

Among the 270 participants with respiratory virus-specific IgG levels measured both during childhood in EGEA1 and early-adulthood in EGEA2 (about 11 years later), the specific levels of IgG significantly decreased over time for each RV species, with a lower magnitude for RV-B as compared to RV-A and RV-C (**Supplementary Figure S11**). These results were similar after adjustment for age at baseline, sex, BMI, tobacco smoke exposure, season of blood sampling, allergic sensitization and ever-asthma (**Table 3**). A similar trend was observed for RSV (p=0.09) (**Table 3**).

**Table 3:** Annual changes of RSV and RV-specific IgG levels in individuals with data both in childhood and adulthood (n=270).

	RSV		RV-A		RV-B		RV-C	
	beta [95%CI]	p	beta [95%CI]	p	beta [95%CI]	p	beta [95%CI]	p
Change in 1 year	-0.02 [-0.04; 0.00]	0.09	-0.04 [-0.06; -0.03]	**8.98E-08**	-0.02 [-0.04; 0.00]	**0.01**	-0.05 [-0.06; -0.03]	**8.93E-10**

RSV, respiratory syncytial virus; RV, rhinovirus.

Beta were estimated from linear mixed regression models for repeated data (random effect on individuals) with IgG level as dependent variable and time between the two surveys (year), age at first survey (continuous), sex, BMI (continuous), season of blood sampling (4 seasons), tobacco (passive in EGEA1, active in EGEA2), allergic sensitization, ever-asthma as predictors.Bold values correspond to significant (p<0.05) p-values.

## 4 Discussion

To the best of our knowledge, this study is the first to investigate the associations of personal and seasonal factors with IgG levels to RSV and RV viruses in a large population of children and adults. Results showed that an older age was associated with higher RV-specific IgG levels in children (aged between 2-16 years) and lower RV-specific IgG level in adults (aged between 18-79 years) while it was associated with higher RSV-specific level in both children and adults. RV-specific IgG levels were lower in males and in participants with higher BMI both in children and adults, while RSV-specific IgG levels were not associated with sex or BMI. Higher levels of RSV- and RV-specific IgG were found during winter, spring and autumn in adults while no significant association with season was observed for children. Current smoking, and to a lesser extent former smoking, were associated with higher IgG levels for RSV- and all RV species in adults whereas passive smoking among children was not associated with RSV- and RV-specific IgG levels. Overall, these results suggest that different determinants might influence the immune response to respiratory viruses, according to the respiratory virus (RSV or RV) and the age (in children or in adults).

### 4.1 Interpretation of results and comparison with previous findings

In our study, the effect of age on the respiratory virus-specific IgG levels varied between RSV and RV and between children and adults, indicating, for the first time, different patterns of virus specific immune responses over the course of life. Regarding RV, our results indicate that IgG levels were higher (RV-B) or remained stable (RV-A and -C) over time during childhood, and were lower with age from adolescents/early adulthood, while RSV IgG levels seemed to increase with age both in childhood and in adulthood. Previous studies in the literature were restricted to children and led to contradictory results regarding the effect of age on RV-specific IgG responses, with studies showing a negative association (15), a positive association (24) or no association (14). These studies were based on relatively small sample sizes (range: 120-300) and were often based on PCR data which do not allow to draw conclusion regarding immune responses in the studied populations thus limiting the comparability between studies. To the best of our knowledge, there are no studies investigating the associations between RSV- and RV-specific immune response among adults.

Regarding sex, our results showing higher RV-specific IgG levels in women than in men confirm the existing knowledge reporting higher innate and adaptative immune response in women than in men for various viruses (25–27). Nevertheless, among adults, an increase in age was associated with a more marked reduction in RV-specific IgG levels among females; whereas all other associations between personal or seasonal factors and RSV- and RV-specific IgG levels were generally stronger among males. Further studies are needed to identify the respective effects of hormones, of gender differences in environmental exposures and lifestyle factors and of genetic factors in the sex-specific immune responses.

Our results suggest that higher BMI was associated with lower levels of RV-specific IgG in both children and adults, which is in line with previous observations. It is well known that obesity is a risk factor for chronic diseases, and it seems to be often linked to impaired immunity (28). Indeed, in obese children and adults, excessive mass of adipose tissues impairs proper development and activity of immune cells (29), possibly through an anti-inflammatory mechanism (30). However, no clear connection between obesity and antibody response to respiratory viruses is established.

Regarding smoking, our results did not provide evidence for an effect of passive smoking on RV- and RSV-specific IgG levels among children, but adults with former smoking and, to a greater extent, with current smoking had higher IgG levels against RSV and all RV species. Similarly to our results, one study performed among 217 children from birth to one year of age did not find any association between RSV IgG level and passive smoke/smoking during pregnancy (10). Among adults, a former study did not report any association between tobacco smoking and RSV immune responses, assessed by complement fixation test (11). More recently, a study performed among 245 adults, including two third of HIV+ patients, identified a positive association between current smoking and RSV specific IgG, independently from HIV status (12). Interestingly, a positive effect of smoking on respiratory-virus specific IgG responses is supported by *in vitro* studies that demonstrated that ICAM1, a major epithelial airway receptor for RV in both large and small airways, is upregulated in smokers, resulting in an increased vulnerability of smokers to RV infections (31, 32). Furthermore, tobacco smoke and RV infection may impair the barrier function of respiratory epithelium (33).

RSV and RV can be detected throughout the year, but highest prevalence and most of illness related to these viruses occur during winter (22, 34, 35). Accordingly, in our study we observed higher levels of RSV- specific IgG in all seasons as compared with summer in adults. Since we did not have information when virus infections had occurred in the study population we could only report about the virus-specific antibody levels at different seasons. Until now, several studies investigated the effect of the season of birth on RSV specific IgG responses in young children (10, 36) but, no study focused on the season of blood sampling and respiratory virus-specific IgG responses. This seasonal variations of immune response may be attributable to varying RV and RSV incidences during the year (37–40).

### 4.2 Strengths and weaknesses

Our study has several strengths. First, it is based on a large population of children and adults covering a wide range of ages (range: 2-80), with detailed assessment of both RSV- and RV-specific IgG levels and personal factors. Moreover, this population includes a subpopulation of individuals with respiratory viruses IgG assessment in both childhood and early adulthood allowing to specifically characterize change in IgG levels at the individual level over time. A validated micro-array technique was used to assess IgG levels. Then, different antigens were studied, related to two different respiratory viruses, RSV and RV. For RV, the analyses were based on micro-arrayed VP1 N-terminal peptides from a panel of the most representative RV strains covering the three genetic species. In this study, measurements of cumulative RV-A, RV-B and RV-C specific antibody levels (i.e., sums of RV-A-, RV-B-, or RV-C peptide derived IgG levels) could be performed which allow to calculate the sum of species-specific IgG levels which are indicative of previous virus encounter and mounting of specific IgG antibodies as described in (41). Then, we have carefully chosen peptides which are rather specific for RV-A and RV-C. One can therefore assume, that the sums of IgG levels to the peptides selected for RV-A, RV-B and RV-C are indeed indicative for RV-A, RV-B and RV-C species-specific IgG antibody responses. Finally, EGEA1 and EGEA2 blood samples were collected twelve years apart but serum IgG antibodies are stable over time, not sensitive to serum storage duration (42).

We acknowledge that our study also suffers from some limitations. First, we did not have any information about the number of infections or the time at which viral infection occurs. Consequently, we were unable to assess the time elapsed between the last viral infection and the time of blood sampling. In the population with two follow-ups, the lack of this information prevents us from concluding on the association of personal or seasonal factors with the persistence of IgG levels following exposure to the virus. Secondly, some personal factors, like genetic factors which could interact with personal factors, were not considered in this analysis, and should deserve further investigation but are out of the scope of the present study. Furthermore, the large majority of our population is Caucasian, and generalization of our results to other ethnic groups cannot be done, but allows analyzing a homogenous population, therefore limiting risk for confounding bias. Additionally, one has to consider that a percentage of subjects (≥ 55%) had an allergic sensitization. However, analyses performed in the subpopulation of individuals with and without allergic sensitization showed similar estimates than those reported in the whole population. Therefore, our results were not influenced by a collider bias due to a high prevalence of individuals with allergic sensitization. Finally, given the EGEA study design, asthma could be considered as a collider bias and may distort the association investigated (43). However, the fact that all the patterns of associations reported in this study were stable regardless of the asthma status supports the absence of collider bias in our results.

### 4.3 Conclusion

Our study investigated for the first time the associations between several personal factors and RSV- and RV-specific antibody levels in a large population covering a wide age range. In children, older age and female sex were associated with higher RV-specific IgG levels and higher BMI was associated with lower RV-specific IgG levels, while no personal determinants were associated with RSV-specific IgG levels. In adults, age (older age for RSV, younger age for RV), active smoking and all seasons except summer were associated with higher RSV- and RV- specific IgG levels. Additionally, female sex was associated with higher RV- specific IgG levels and higher BMI was associated with lower RV-specific IgG levels among adults. These results suggest that personal determinants of IgG responses to respiratory viruses might differ between children and adults and depending of the type of respiratory virus.

## Data availability statement

The datasets presented in this article are not readily available because data are confidential. Requests to access the datasets should be directed to valerie.siroux@univ-grenoble-alpes.fr.

## Ethics statement

The studies involving humans were approved by Cochin Royal Hospital, Paris for EGEA1 and Necker-Enfants Malades Hospital, Paris for EGEA2. The studies were conducted in accordance with the local legislation and institutional requirements. Written informed consent for participation in this study was provided by the participants’ legal guardians/next of kin. Analysis of pseudonymized samples was performed at the Medical University of Vienna with permission from the Ethics committee of the Medical university of Vienna EK (EK1721/2014).

## Author contributions

AG: Conceptualization, Methodology, Resources, Software, Writing – original draft. KN: Conceptualization, Methodology, Resources, Software, Writing – original draft. MM: Conceptualization, Methodology, Resources, Software, Writing – original draft. AB: Resources, Writing – review & editing. RVa: Resources, Writing – review & editing. BL: Resources, Writing – review & editing. OD: Resources, Writing – review & editing. NL: Resources, Validation, Writing – review & editing. TS: Resources, Writing – review & editing. MB: Resources, Writing – review & editing. KB: Resources, Writing – review & editing. PE: Resources, Writing – review & editing. RVe: Resources, Writing – review & editing. RN: Resources, Writing – review & editing. JB: Resources, Writing – review & editing. EB: Resources, Writing – review & editing. RuV: Conceptualization, Methodology, Resources, Software, Writing – original draft. VS: Conceptualization, Methodology, Resources, Software, Writing – original draft.
