# Supplementary material for: Determinants of immunoglobulin G responses to respiratory syncytial virus and rhinovirus in children and adults
Source: Front Immunol. 2024 Mar 4;15:1355214. doi: 10.3389/fimmu.2024.1355214 (PMC10945029; doi:10.3389/fimmu.2024.1355214)
Supplement: Supplementary file 1 [file DataSheet_1.docx]

**Determinants of Immunoglobulin G responses to respiratory syncytial virus and rhinovirus in children and adults**

***Supplementary Material***

Alicia Guillien, Katarzyna Niespodziana, Marion Mauclin, Anne Boudier, Raphäelle Varraso, Bénédicte Leynaert, Orianne Dumas, Nicole Le Moual, Thomas Schlederer, Maja Bajic, Kristina Borochova, Peter Errhalt, Raphael Vernet, Rachel Nadif, Jean Bousquet, Emmanuelle Bouzigon, Rudolf Valenta, Valérie Siroux

Plan

[Methods 2](#_Toc157776167)

[Measurement of virus-specific IgG using micro-array technology 2](#_Toc157776168)

[Calibration of virus-specific IgG data 2](#_Toc157776169)

[Correction of virus-specific IgG data for the background level 3](#_Toc157776170)

[Results 4](#_Toc157776171)

[Table S1. Amino acid sequences of the micro-arrayed RV-derived synthetic VP1 peptides and RSV-G protein 4](#_Toc157776172)

[Table S2. List of control allergens spotted onto the microarray. 6](#_Toc157776173)

[Table S3. Estimated associations between personal factors and RSV- and RV-specific IgG levels in children (EGEA1, n=530) and adults (EGEA2, n=1241) from multiple regression models 7](#_Toc157776174)

[Supplemental figures 9](#_Toc157776175)

# Methods

## Measurement of virus-specific IgG using micro-array technology

For the determination of virus-specific IgG antibodies, microarrays were processed as previously described (Niespodziana *et al.,* 2018, Niespodziana *et al*., 2021). Serum samples were diluted in two steps using an ImmunoCAP® Specific IgA/IgG Sample Diluent (Phadia, Uppsala, Sweden) to a final dilution of 1:300. Twenty slides per analysis run, each containing six arrays, were washed with phosphate-buffered saline with 0.1% Tween 20 (PBS/Tween) and dried by centrifugation using a Sigma 2–7 centrifuge and MTP-11113 rotor (both Sigma Laborzentrifugen GmbH, Osterode am Harz, Germany) for 2 min at 159 x g Thirty µl of 1:300 diluted sera, a calibrator and sample diluent were applied onto each microarray and incubated for 2 hours at gentle rocking (6 times per minute) at room temperature (RT) (Biometra, Jena, Germany). Afterwards slides were washed again with PBS/Tween, dried by centrifugation and incubated with 30 µl/per array of the DyLight 550 (Pierce, Rockford, IL, USA) labelled anti-human IgG (1.8 µg/mL) (Jackson ImmunoResearch Laboratories, West Grove, PA, USA) for 30 min at RT. After further rinsing, washing with PBS/Tween and drying by centrifugation as described above, microarrays were scanned with a confocal PowerScanner from Tecan Grödig, Austria, using 30% of gain (i.e., photomultiplier (PMT)) and 10% of laser power.

Scanned images were analysed using the Mappix software (Innopsys, Carbonne, France). Fluorescence intensities (FI) of three replicated spots (i.e., raw data) were exported to an Excel file (Microsoft Corporation, Redmond, Washington, USA) and the median value of triplicate measurements was calculated.

## Calibration of virus-specific IgG data

Raw data were divided in 21 assays, each containing an "IgG calibrator" sample and a "Sample Diluent (SD)" sample.

We performed the calibration of raw data using the following procedure:

- within each assay: for a given epitope, the "calibrator-SD" difference is calculated, i.e. [median value of IgG calibrator] - [ median value of SD]
- for a given epitope, we calculated the average of the differences, i.e. the average of "calibrator-SD" over the 21 assays
- we then calculated, for each assay, the following linear regression: "calibrator-SD" as a function of the "average of the differences", with the epitope as the unit of study. We thus performed 21 linear regressions (1 for each assay of n slides, n going from 5 to 20) and obtained 21 regression coefficients (i.e. slope of the regression line)

For a peptide P measured for a subject S included in the assay N, the calibrated value of the peptide P of this subject S corresponds to the median IgG value of the 3 observed spots of the peptide P divided by the beta (= slope of the regression line) of the calibration of the assay N (N ranging from 1 to 21).

## Correction of virus-specific IgG data for the background level

In order to determine the analytical sensitivity of each antigen, the background signals of all sample diluent repetitive measurements (n = 21) were calculated as a mean FI values + 3 SD for each antigen (corrected data). Values below this background signal were fixed to zero. For RV-specific IgG data, summary RV-A, RV-B and RV-C variables were calculated by the sum of IgG levels to 18, 9 and 10 specific peptides respectively, as listed in Table S1.

# Results

## **Table S1**. Amino acid sequences of the micro-arrayed RV-derived synthetic VP1 peptides and RSV-G protein

| **Peptide** | **Species** | **Strain** | **Amino acid sequence** | **Position^†^** | **Length [aa]**^‡^ | **Molecular weight [Dalton]** | **pI** | **GenBank accession number** |
| --- | --- | --- | --- | --- | --- | --- | --- | --- |
| ***RV-A species*** | | | | | | | | |
| **01p1** | RV-A | 1B | NPVENYIDEVLNEVLVVPNIKESHHTTSNSAPLLDAAETG | 1-40 | 40 | 4331 | 4.24 | AAQ19856 |
| **02p1** | RV-A | 2 | NPVENYIDEVLNEVLVVPNINSSNPTTSNSAPALDAAETG | 1-40 | 40 | 4169 | 3.34 | AAQ19857 |
| **08p1** | RV-A | 8 | NPIEQFTEAVLNEVLVVPNTQASNGSIANSAPALDAAETG | 1-40 | 40 | 4053 | 3.45 | ACK37369 |
| **11p1** | RV-A | 11 | NPVEDYVDGILNEVLVVPNIKESQATTSNSAPALDAAETG | 1-40 | 40 | 4143 | 3.66 | AAQ19866 |
| **12p1** | RV-A | 12 | NPVERYVDEVLNEVLVVPNINKSNGQLSNAAPALDAAETG | 1-40 | 40 | 4222 | 4.08 | AAQ19867 |
| **16p1** | RV-A | 16 | NPVERYVDEVLNEVLVVPNINESHPTTSNAAPVLDAAETG | 1-40 | 40 | 4275 | 4.00 | AAQ19871 |
| **18p1** | RV-A | 18 | NPVENYIDEVLNEVLVVPNVNESHAITSNSAPALDAAETG | 1-40 | 40 | 4207 | 3.71 | AAQ19873 |
| **19p1** | RV-A | 19 | NPVEKYVDTILNEVLVVPNINESHPSTSNAAPALDAAETG | 1-40 | 40 | 4158 | 4.08 | ACK37375 |
| **20p1** | RV-A | 20 | NPVERYTEAILNEVLVVPNITSSNSQTSNAAPALDAAETG | 1-40 | 40 | 4158 | 3.91 | ACK37376 |
| **25p1** | RV-A | 25 | NPIENYVDQVLNEVLVVPNIKESHPSTSNSAPILDAAETG | 1-40 | 40 | 4276 | 4.08 | ACK37379 |
| **28p1** | RV-A | 28 | NPVEKYTEALLNEVLVVPNINPSNAQTTNAAPALDAAETG | 1-40 | 40 | 4151 | 3.91 | AAQ19883 |
| **29p1** | RV-A | 29 | NPVENYVDEVLNEVLVVPNIRESHPSTSNSAPILDAAETG | 1-40 | 40 | 4291 | 4.00 | ACK37381 |
| **43p1** | RV-A | 43 | NPVENYVDEILNQVLVVPNTVESHSTTSNAAPALDAAETG | 1-40 | 40 | 4181 | 3.77 | ACK37387 |
| **45p1** | RV-A | 45 | NPVEQFAEAVLDQVLVVPNTRPSDGLIANSAPALDAAETG | 1-40 | 40 | 4091 | 3.71 | ACK37388 |
| **59p1** | RV-A | 59 | NPVENYVNDVLNEVLVVPNIQESHPTTSNAAPALDAAETG | 1-40 | 40 | 4204 | 3.77 | AAQ19914 |
| **68p1** | RV-A | 68 | NPVEKYTEAVLNEVLVVPNIPASNTQTSNAAPALDAAETG | 1-40 | 40 | 4110 | 3.91 | ACK37406 |
| **78p1** | RV-A | 78 | NPVEEYVDQVLNEVLVVPNIKESKPQSSNSAPVLDAAETG | 1-40 | 40 | 4281 | 4.00 | ACK37439 |
| **89p1** | RV-A | 89 | NPVENYIDSVLNEVLVVPNIQPSTSVSSHAAPALDAAETG | 1-31 | 31 | 3293 | 4.13 | AAQ19944 |
| ***RV-B species*** | | | | | | | | |
| **04p1** | RV-B | 4 | GLGEDLMEVIVDKTHQTLASVKSDSKHTQKVPALTANETG | 1-40 | 40 | 4250 | 5.41 | AAQ19859 |
| **05p1** | RV-B | 5 | GLEDDLVEVIVDKAQQTLASIKSDSKHTQKVPSLTANETG | 1-40 | 40 | 4267 | 4.65 | AAQ19860 |
| **14p1** | RV-B | 14 | GLGDELEEVIVEKTKQTVASISSGPKHTQKVPILTANETG | 1-40 | 40 | 4206 | 5.06 | AAQ19869 |
| **17p1** | RV-B | 17 | GFEDELEEVVIDKMKQVTASSQSGPKHTQKVPALSANETG | 1-40 | 40 | 4287 | 4.75 | AAQ19872 |
| **27p1** | RV-B | 27 | GLGEELEEVIVDKAKQTIASVSSNSKHTQKVPTLSASETG | 1-40 | 40 | 4170 | 5.06 | ACK37442 |
| **69p1** | RV-B | 69 | GLGEELEEVVIDKMKQVTASVQSGSKHTQKVPALSASETG | 1-40 | 40 | 4170 | 5.06 | ACK37407 |
| **70p1** | RV-B | 70 | GFEGELEEVVIDKMKQVTASSQSGPKYTQKVPALSANETG | 1-40 | 40 | 4255 | 4.72 | AAQ19925 |
| **84p1** | RV-B | 84 | GLEDVLEEVIVDKAKQTIASINSNSKYTQQVPTLSASETG | 1-40 | 40 | 4265 | 4.36 | AAQ19939 |
| **86p1** | RV-B | 86 | GLGDELEEVIVEKTKQTLASVATGSKYTQKVPSLSANETG | 1-40 | 40 | 4180 | 4.72 | ACK37420 |
| ***RV-C species*** | | | | | | | | |
| **YPp1** | RV-C | C_YP | NPVEDYIDKVVDTVLQVPNTQPSGPQHSIQPSALGAMEIG | 1-40 | 40 | 4246 | 4.10 | YP_001552435 |
| **QPMp1** | RV-C | C_QPM | NPVEEFVEHTLKEVLVVPDTQASGPVHTTKPQALGAVEIG | 1-40 | 40 | 4238 | 4.68 | ABP38410 |
| **c025p1** | RV-C | C_025 | NPVEQFVDNVLEEVLVVPNTQPSGPIHTTKPTALSAMEIG | 1-40 | 40 | 4273 | 4.25 | ABU62849 |
| **C1p1** | RV-C | C1 | NPVEEFVDHTLQKVMAVPDTQASGPTHTNKPQTLGALEIG | 1-40 | 40 | 4272 | 4.87 | ABQ16587 |
| **C2p1** | RV-C | C2 | NPVDNFVDEVLKEVLVVPDTKPSGPTHTVKPTVLNAMEIG | 1-40 | 40 | 4301 | 4.58 | AET25086 |
| **C7p1** | RV-C | C7 | NPVEKFIDQTLEEVLVVPDTQASGPVHTTKPQTLGALEIG | 1-40 | 40 | 4273 | 4.35 | AER92557 |
| **C8p1** | RV-C | C8 | NNDPVDGFVHEVLNEVVVVPDTKPSGPQHTTKPSALGAMEIG | 1-42 | 42 | 4397 | 4.57 | AFD64766 |
| **C11p1** | RV-C | C11 | NNDDLVENFVESTLKEVLVVPDTKPSGPQHTTKPSILGAMEIG | 1-43 | 43 | 4621 | 4.43 | AET25079 |
| **C26p1** | RV-C | C26 | NPVEEYIDETLKEVLVVPNTKSTGPTHTTKPSALGAMEIG | 1-40 | 40 | 4267 | 4.70 | AFM84629 |
| **C35p1** | RV-C | C35 | NPVETFTEEVLKEVLVVPNTQPSGPSHTVRPTALGALEIG | 1-40 | 40 | 4228 | 4.64 | AEM76814 |
| ***RSV*** | | | | | | | | |
| **A2-G** | RSV | A2 | HKVTPTTAIIQDATSQIKNTTPTYLTQNPQLGISPSNPSEITSQITTILASTTPGVKSTLQSTTVKTKN  TTTTQTQPSKPTTKQRQNKPPSKPNNDFHFEVFNFVPCSICSNNPTCWAICKRIPNKKPGKKTTTK  PTKKPTLKTTKKDPKPQTTKSKEVPTTKPTEEPTINTTKTNIITTLLTSNTTGNPELTSQMETFHSTS  SEGNPSPSQVSTTSEYPSQPSSPPNTPRQ | 66-298 | 232 | 25251 | 9.87 | P03423 |

^†^Position denotes the corresponding region of the VP1 protein.

^‡^Length: number of amino acids, aa.

## **Table S2**. List of control allergens spotted onto the microarray.

| **Microarray component** | **Allergen** | **Name** |
| --- | --- | --- |
| QC1 | Bovine Serum Albumin | Bos d 6 |
| QC2 | ß-Lactoglobulin | Bos d 5 |
| QC3 | Lactoferrin Bovine Milk | Bos d lfn |
| QC4 | Casein | Bos d 8 |
| QC5 | Ovatransferrin | Gal d 3 |
| QC6 | Chicken Egg Albumin | Gal d 2 |
| QC7 | Chicken Egg Lysozyme | Gal d 4 |
| QC8 | Trypsin Inhibitor Chicken Egg | Gal d 1 |

## **Table S3**. Estimated associations between personal factors and RSV- and RV-specific IgG levels in children (EGEA1, n=530) and adults (EGEA2, n=1241) from multiple regression models

|  | **EGEA1 (n=530)** | |  | **EGEA2 (n=1241)** | |
| --- | --- | --- | --- | --- | --- |
|  | **beta [95% CI]** | **p** |  | **beta [95% CI]** | **p** |
| **RSV G protein** |  |  |  |  |  |
| Age^†^ | 0.11 [0.02 ; 0.21] | **0.02** |  | 0.18 [0.11 ; 0.24] | **2.44E-07** |
| Sex (Female vs Male) | 0.07 [-0.09 ; 0.23] | 0.37 |  | 0.06 [-0.05 ; 0.17] | 0.31 |
| BMI^†^ | -0.04 [-0.14 ; 0.06] | 0.41 |  | -0.04 [-0.10 ; 0.03] | 0.24 |
| Season |  |  |  |  |  |
| January-March | 0.17 [-0.06 ; 0.40] | 0.15 |  | 0.18 [0.00 ; 0.36] | **0.05** |
| April-June | -0.02 [-0.27 ; 0.22] | 0.85 |  | 0.27 [0.10 ; 0.44] | **2.21E-03** |
| July-September | 0.00 |  |  | 0.00 |  |
| October-December | -0.17 [-0.40 ; 0.06] | 0.15 |  | 0.20 [0.03 ; 0.38] | **0.02** |
| Passive smoking (Yes vs No) | 0.00 [-0.18 ; 0.17] | 0.98 |  | - |  |
| Tobacco status |  |  |  |  |  |
| Non-smoker | - |  |  | 0.00 |  |
| Ex-smoker | - |  |  | 0.08 [-0.06 ; 0.22] | 0.25 |
| Active-smoker | - |  |  | 0.18 [0.03 ; 0.33] | **0.02** |
| **RV-A** |  |  |  |  |  |
| Age^†^ | 0.05 [-0.03 ; 0.12] | 0.26 |  | -0.24 [-0.30 ; -0.18] | **1.29E-17** |
| Sex (Female vs Male) | 0.16 [0.03 ; 0.29] | **0.02** |  | 0.10 [0.00 ; 0.19] | **0.05** |
| BMI^†^ | -0.12 [-0.20 ; -0.04] | **0.05** |  | -0.06 [-0.12 ; -0.01] | **0.03** |
| Season |  |  |  |  |  |
| January-March | 0.19 [-0.01 ; 0.39] | 0.06 |  | 0.23 [0.07 ; 0.38] | **3.86E-03** |
| April-June | 0.16 [-0.06 ; 0.37] | 0.15 |  | 0.21 [0.06 ; 0.35] | **4.93E-03** |
| July-September | 0.00 |  |  | 0.00 |  |
| October-December | 0.06 [-0.14 ; 0.26] | 0.55 |  | 0.27 [0.12 ; 0.42] | **3.80E-04** |
| Passive smoking (Yes vs No) | 0.11 [-0.04 ; 0.25] | 0.15 |  | - |  |
| Tobacco status |  |  |  |  |  |
| Non-smoker | - |  |  | 0.00 |  |
| Ex-smoker | - |  |  | 0.08 [-0.03 ; 0.20] | 0.17 |
| Active-smoker | - |  |  | 0.27 [0.14 ; 0.39] | **3.35E-05** |
| **RV-B** |  |  |  |  |  |
| Age^†^ | 0.12 [0.04 ; 0.21] | **2.78E-03** |  | -0.20 [-0.26 ; -0.13] | **1.15E-09** |
| Sex (Female vs Male) | 0.15 [0.01 ; 0.28] | **0.03** |  | 0.13 [0.02 ; 0.23] | **0.02** |
| BMI^†^ | -0.07 [-0.16 ; 0.01] | 0.08 |  | -0.07 [-0.13 ; -0.01] | **0.02** |
| Season |  |  |  |  |  |
| January-March | 0.19 [-0.01 ; 0.40] | 0.06 |  | 0.14 [-0.04 ; 0.31] | 0.12 |
| April-June | 0.07 [-0.15 ; 0.30] | 0.52 |  | 0.19 [0.03 ; 0.35] | **0.02** |
| July-September | 0.00 |  |  | 0.00 |  |
| October-December | 0.14 [-0.07 ; 0.35] | 0.19 |  | 0.21 [0.04 ; 0.38] | **0.02** |
| Passive smoking (Yes vs No) | -0.01 [-0.17 ; 0.15] | 0.92 |  | - |  |
| Tobacco status |  |  |  |  |  |
| Non-smoker | - |  |  | 0.00 |  |
| Ex-smoker | - |  |  | 0.13 [0.00 ; 0.26] | **0.04** |
| Active-smoker | - |  |  | 0.36 [0.22 ; 0.50] | **5.43E-07** |
| **RV-C** |  |  |  |  |  |
| Age^†^ | 0.03 [-0.05 ; 0.11] | 0.51 |  | -0.23 [-0.28 ; -0.17] | **8.30E-16** |
| Sex (Female vs Male) | 0.17 [0.03 ; 0.31] | **0.02** |  | 0.08 [-0.01 ; 0.18] | 0.08 |
| BMI^†^ | -0.09 [-0.17 ; 0.00] | **0.04** |  | -0.07 [-0.12 ; -0.01] | **0.01** |
| Season |  |  |  |  |  |
| January-March | 0.14 [-0.06 ; 0.34] | 0.19 |  | 0.21 [0.05 ; 0.36] | **7.83E-03** |
| April-June | 0.06 [-0.16 ; 0.27] | 0.60 |  | 0.22 [0.08 ; 0.36] | **2.52E-03** |
| July-September | 0.00 |  |  | 0.00 |  |
| October-December | 0.06 [-0.15 ; 0.26] | 0.59 |  | 0.21 [0.06 ; 0.35] | **6.66E-03** |
| Passive smoking (Yes vs No) | 0.14 [0.00 ; 0.29] | 0.06 |  | - |  |
| Tobacco status |  |  |  |  |  |
| Non-smoker | - |  |  |  |  |
| Ex-smoker | - |  |  | 0.03 [-0.09 ; 0.14] | 0.64 |
| Active-smoker | - |  |  | 0.23 [0.11 ; 0.36] | **2.39E-04** |

Beta were estimated from linear mixed regression models for repeated data (random effect on family) with IgG level as dependent variable and age (continuous), sex, BMI (continuous), season of sampling (4 seasons) and tobacco (passive smoking at EGEA1, active smoking at EGEA2) as predictors. All models were adjusted for allergic sensitization and ever-asthma and were applied on the multiple imputed dataset.

EGEA: Epidemiological study on the Genetics and Environment of Asthma; RSV: respiratory syncytial virus; RV: human rhinovirus; CI: confidence interval; BMI: body mass index.

^†^Age and BMI were standardized; SD of age EGEA1: 2.85 years, EGEA2: 16.7 years; SD of BMI EGEA1: 2.91 kg/m2, EGEA2: 4.25 kg/m2")

# Supplemental figures

**Figure S1. Workflow of the study**

IgG: Immunoglobulin G; RV: rhinovirus; RSV: respiratory syncytial virus.

**Figure S2. Distribution of RSV and RV-specific peptides IgG data among children (EGEA1, n=531) and adults (EGEA2, n=1241)**

RSV: respiratory syncytial virus; RV: rhinovirus; EGEA: Epidemiological study on the Genetics and Environment of Asthma.

**Figure S3. Correlation matrix of RSV- and RV-specific IgG levels in children (EGEA1, n=531) and adults (EGEA2, n=1241)**

Demonstrated is a color-mapped correlation matrix containing Pearson coefficients (r) obtained for IgG levels measured in children (left matrix) and adults (right matrix). Red cell represents a strong correlation while a white cell represents no correlation.

RSV: respiratory syncytial virus; RV: rhinovirus; EGEA: Epidemiological study on the Genetics and Environment of Asthma.

**Figure S4. Associations of RSV- and RV-specific IgG levels with personal determinants, season of sampling in children (n=470) and adults (n=1109) without respiratory infections in the three (four for EGEA2 population) weeks prior to blood collection**

Multivariable mixed linear regression models were used to estimate beta and corresponding 95% CIs for virus-specific (RSV/RV) antibody levels expressed as z-scores associated with personal determinants and the seasons of sampling. Error bars represent 95% CIs.

EGEA: Epidemiological study on the Genetics and Environment of Asthma; RSV: respiratory syncytial virus; RV: rhinovirus; BMI: body mass index; Jan-Mar: January-March; Apr-Jun: April-June; Jul-Sep: July-September; Oct-Dec: October-December.

Beta were estimated from linear mixed regression models for repeated data (random effect on family) with IgG level as dependent variable and age (continuous), sex, BMI (continuous), season of sampling (4 seasons), passive smoking, ever-asthma and allergic sensitization as predictors.

*age and BMI were standardized (divided by their Standard deviation (SD)), thus beta are reported for 1SD increased. Standard deviation (SD) of age at EGEA1: 2.85 years; SD of BMI at EGEA1: 2.91 kg/m^2^. Standard deviation (SD) of age at EGEA2: 16.73 years; SD of BMI at EGEA2: 4.25 kg/m^2^.

**Figure S5. Associations of RSV- and RV-specific IgG levels with personal determinants, season of sampling in boys (n=303) and girls (n=227) in the EGEA1 population of children**

Multivariable mixed linear regression models were used to estimate beta and corresponding 95% CIs for virus-specific (RSV/RV) antibody levels expressed as z-scores associated with personal determinants and the seasons of sampling. Error bars represent 95% CIs.

EGEA: Epidemiological study on the Genetics and Environment of Asthma; RSV: respiratory syncytial virus; RV: rhinovirus; BMI: body mass index; Jan-Mar: January-March; Apr-Jun: April-June; Jul-Sep: July-September; Oct-Dec: October-December.

Beta were estimated from linear mixed regression models for repeated data (random effect on family) with IgG level as dependent variable and age (continuous), BMI (continuous), season of sampling (4 seasons), passive smoking, ever-asthma and allergic sensitization as predictors.

*age and BMI were standardized (divided by their Standard deviation (SD)), thus beta are reported for 1SD increased. Standard deviation (SD) of age at EGEA1: 2.85 years; SD of BMI at EGEA1: 2.91 kg/m^2^.

**Figure S6. Associations of RSV- and RV-specific IgG levels with personal determinants, season of sampling in men (n=595) and women (n=646) in the EGEA2 population of adults**

Multivariable mixed linear regression models were used to estimate beta and corresponding 95% CIs for virus-specific (RSV/RV) antibody levels expressed as z-scores associated with personal determinants and the seasons of sampling. Error bars represent 95% CIs.

EGEA: Epidemiological study on the Genetics and Environment of Asthma; RSV: respiratory syncytial virus; RV: rhinovirus; BMI: body mass index; Jan-Mar: January-March; Apr-Jun: April-June; Jul-Sep: July-September; Oct-Dec: October-December.

Beta were estimated from linear mixed regression models for repeated data (random effect on family) with IgG level as dependent variable and age (continuous), BMI (continuous), season of sampling (4 seasons), passive smoking, ever-asthma and allergic sensitization as predictors.

*age and BMI were standardized (divided by their Standard deviation (SD)), thus beta are reported for 1SD increased. Standard deviation (SD) of age at EGEA2: 16.73 years; SD of BMI at EGEA2: 4.25 kg/m^2^.

**Figure S7. Associations of RSV- and RV-specific IgG levels with personal determinants, season of sampling in children without ever-asthma (n=252) and in children with ever-asthma (n=274)**

Multivariable mixed linear regression models were used to estimate beta and corresponding 95% CIs for virus-specific (RSV/RV) antibody levels expressed as z-scores associated with personal determinants and the seasons of sampling. Error bars represent 95% CIs.

EGEA: Epidemiological study on the Genetics and Environment of Asthma; RSV: respiratory syncytial virus; RV: rhinovirus; BMI: body mass index; Jan-Mar: January-March; Apr-Jun: April-June; Jul-Sep: July-September; Oct-Dec: October-December.

Beta were estimated from linear mixed regression models for repeated data (random effect on family) with IgG level as dependent variable and age (continuous), sex, BMI (continuous), season of sampling (4 seasons), passive smoking and allergic sensitization as predictors.

*age and BMI were standardized (divided by their Standard deviation (SD)), thus beta are reported for 1SD increased. Standard deviation (SD) of age at EGEA1: 2.85 years; SD of BMI at EGEA1: 2.91 kg/m^2^.

**Figure S8. Associations of RSV- and RV-specific IgG levels with personal determinants, season of sampling in adults without ever-asthma (n=743) and in adults with ever-asthma (n=498)**

Multivariable mixed linear regression models were used to estimate beta and corresponding 95% CIs for virus-specific (RSV/RV) antibody levels expressed as z-scores associated with personal determinants and the seasons of sampling. Error bars represent 95% CIs.

EGEA: Epidemiological study on the Genetics and Environment of Asthma; RSV: respiratory syncytial virus; RV: rhinovirus; BMI: body mass index; Jan-Mar: January-March; Apr-Jun: April-June; Jul-Sep: July-September; Oct-Dec: October-December.

Beta were estimated from linear mixed regression models for repeated data (random effect on family) with IgG level as dependent variable and age (continuous), sex, BMI (continuous), season of sampling (4 seasons), active smoking tobacco and allergic sensitization as predictors.

*age and BMI were standardized (divided by their Standard deviation (SD)), thus beta are reported for 1SD increased. Standard deviation (SD) of age at EGEA2: 16.7 years; SD of BMI at EGEA1: 2.91 kg/m^2^; SD of BMI at EGEA2: 4.25 kg/m^2^.

**Figure S9. Associations of RSV- and RV-specific IgG levels with personal determinants, season of sampling in children without allergic sensitization (n=189) and in children with allergic sensitization (n=331)**

Multivariable mixed linear regression models were used to estimate beta and corresponding 95% CIs for virus-specific (RSV/RV) antibody levels expressed as z-scores associated with personal determinants and the seasons of sampling. Error bars represent 95% CIs.

EGEA: Epidemiological study on the Genetics and Environment of Asthma; RSV: respiratory syncytial virus; RV: rhinovirus; BMI: body mass index; Jan-Mar: January-March; Apr-Jun: April-June; Jul-Sep: July-September; Oct-Dec: October-December.

Beta were estimated from linear mixed regression models for repeated data (random effect on family) with IgG level as dependent variable and age (continuous), sex, BMI (continuous), season of sampling (4 seasons), passive smoking and ever-asthma as predictors.

*age and BMI were standardized (divided by their Standard deviation (SD)), thus beta are reported for 1SD increased. Standard deviation (SD) of age at EGEA1: 2.85 years; SD of BMI at EGEA1: 2.91 kg/m^2^.

**Figure S10. Associations of RSV- and RV-specific IgG levels with personal determinants, season of sampling in adults without allergic sensitization (n=526) and in adults with allergic sensitization (n=648)**

Multivariable mixed linear regression models were used to estimate beta and corresponding 95% CIs for virus-specific (RSV/RV) antibody levels expressed as z-scores associated with personal determinants and the seasons of sampling. Error bars represent 95% CIs.

EGEA: Epidemiological study on the Genetics and Environment of Asthma; RSV: respiratory syncytial virus; RV: rhinovirus; BMI: body mass index; Jan-Mar: January-March; Apr-Jun: April-June; Jul-Sep: July-September; Oct-Dec: October-December.

Beta were estimated from linear mixed regression models for repeated data (random effect on family) with IgG level as dependent variable and age (continuous), sex, BMI (continuous), season of sampling (4 seasons), active smoking tobacco and ever-asthma as predictors.

*age and BMI were standardized (divided by their Standard deviation (SD)), thus beta are reported for 1SD increased. Standard deviation (SD) of age at EGEA2: 16.7 years; SD of BMI at EGEA1: 2.91 kg/m^2^; SD of BMI at EGEA2: 4.25 kg/m^2^.

**Figure S11. Distribution of RSV- and RV-specific IgG levels in individuals with available measurements both in childhood and adulthood (n=270)**

RSV: respiratory syncytial virus; RV: rhinovirus; EGEA: Epidemiological study on the Genetics and Environment of Asthma.
